# Editorial for Special Issue “Phytopathogens: Detection and Control”

**DOI:** 10.3390/microorganisms13122873

**Published:** 2025-12-18

**Authors:** Miłosz Tkaczyk

**Affiliations:** Department of Forest Protection, Forest Research Institute, Braci Leśnej 3, 05-090 Sękocin Stary, Poland; m.tkaczyk@ibles.waw.pl

Plant diseases caused by a wide range of pathogens—including fungi, bacteria, viruses, and fungus-like organisms (oomycetes)—represent one of the most significant threats to global food security, agricultural sustainability, and ecosystem balance. Crop intensification, globalization of trade, and climate change all facilitate the rapid dissemination of pathogenic agents and accelerate their evolution toward greater virulence and resistance to conventional control strategies. Against this backdrop, this Special Issue, “Plant Pathogens: Detection and Control,” highlights recent advances in the identification, monitoring, and management of plant pathogens, with an emphasis on rapid, precise, and field-deployable diagnostic tools.

Recent studies indicate several particularly promising directions of development. Early diagnostics has become a focal area, dominated by molecular approaches based on PCR and its quantitative derivatives, as well as isothermal amplification techniques such as LAMP and RPA, increasingly coupled with CRISPR-Cas systems [1]. These innovations enable pathogen detection prior to the onset of visible symptoms, which is critical for minimizing crop losses. In parallel, biosensors and miniaturized analytical platforms are emerging, utilizing nanomaterials and optoelectronic systems. When integrated with mobile applications, these technologies allow for rapid and relatively low-cost testing directly under field conditions [2]. High-throughput methods—including next-generation sequencing, metagenomics, and digital PCR—are also gaining prominence. These techniques not only facilitate the detection of pathogens that are difficult to identify using traditional approaches but also provide insights into their genetic variability and evolutionary dynamics [3]. Complementary progress is being made in non-invasive imaging methods such as spectroscopy, thermography, and multispectral imaging [4]. Their advantage lies in the ability to monitor plant physiological status prior to symptom expression. Coupled with machine learning algorithms, such methods pave the way for automated classification of threats and forecasting of epidemic dynamics. Together, these approaches bring us closer to fully integrated decision-support systems that combine diagnostic and environmental data, enabling growers to respond rapidly and accurately (Figure 1).

Yet detection is only the first step; sustainable and effective disease control remains equally critical. Increasing attention is being directed toward integrated management strategies that combine conventional chemical control with biological antagonists, biofungicides, resistant cultivars, and nanomaterial-based technologies [5]. Such multifaceted approaches reduce selection pressure, limit pesticide use, minimize environmental impact, and enhance the durability of crop protection. Nonetheless, implementation faces important challenges, including high costs of equipment and reagents, lack of methodological standardization, and difficulties in transferring laboratory-based protocols into field practice. Environmental complexity poses an additional obstacle: many diseases are caused by multiple pathogens simultaneously, while mixed and latent infections complicate the interpretation of diagnostic results [6].

The future of plant pathogens research thus requires continued integration of expertise across disciplines—including molecular biology, bioinformatics, materials engineering, and agricultural sciences—to develop diagnostic and control tools that are sensitive, accessible, and user-friendly. This Special Issue, “Plant Pathogens: Detection and Control,” seeks not only to provide a state-of-the-art overview but also to inspire innovation in plant pathology research. By advancing the understanding of infection mechanisms, improving diagnostic workflows, and fostering sustainable control strategies, the contributions presented here aim to strengthen crop protection and global food security in an environmentally responsible manner.

## Figures and Tables

**Figure 1:**
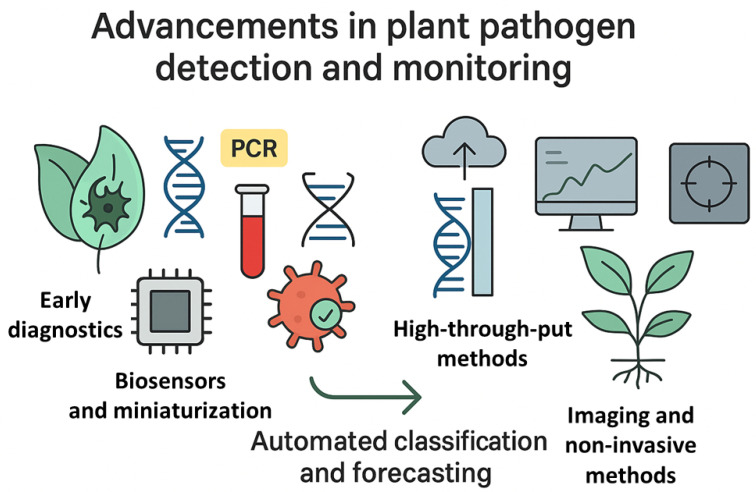
Key directions in the development of plant pathogen diagnostics and monitoring: from early molecular diagnostics, through biosensors and miniaturized technologies, high-throughput methods, and non-invasive imaging, to integrated systems for classification and epidemic forecasting.

## Data Availability

Not applicable.
